# The Animal-foods-environment interface of *Klebsiella pneumoniae* in Germany: an observational study on pathogenicity, resistance development and the current situation

**DOI:** 10.1186/s13567-020-00875-w

**Published:** 2021-02-08

**Authors:** Gamal Wareth, Heinrich Neubauer

**Affiliations:** 1 Friedrich-Loeffler-Institut, Institute of Bacterial Infections and Zoonoses, Naumburger Street 96a, 07743 Jena, Germany; 2 Faculty of Veterinary Medicine, Benha University, Moshtohor, Toukh, 13736 Egypt

**Keywords:** *Klebsiella pneumoniae*, review, occurrence, resistance development, pathogenicity, Germany

## Abstract

*Klebsiella* (*K*.) *pneumoniae* as a multi-drug resistant (MDR) pathogen is an emerging challenge for clinicians worldwide. Virulence factors are capsular antigens, adherence factors, the O-lipopolysaccharide, and siderophores promoting infectivity. Mechanisms of antimicrobial resistance are inactivation of compounds via enzymes, change of membrane permeability, and alteration of the target site of the antimicrobial compound. In addition to environmental resistance, *K. pneumoniae* can survive increasing concentrations of disinfectants, if exposed. This review describes the temporal and spatial distribution of* K. pneumoniae* in the past decades in Germany, with emphases on the development of resistance in the non-human columns of the One-Health concept. In general, *K. pneumoniae* is a neglected pathogen in veterinary and environmental health, and the risk of human infection concerning animal contact and food consumption is barely investigated. Few reports exist (n = 26) on antibiotic resistance of isolates from non-human origin. Multi-drug resistance and extended-spectrum β-lactamase (MDR-ESBL) strains also resistant to carbapenems and antibiotics of the ß-lactam group harbor *bla*CTX-M, *bla*OXA, *bla*TEM, *bla*SHV, *bla*CMY, and PMQR have been found in animals, foods, and the environment. Colistin resistant strains carrying the *mcr*-1 gene were detected in wastewater. The *bla*CTX-M-15 and *bla*OXA-48 genes are the most frequently identified AMR genes in isolates of humans and were also the most predominant ESBL-genes in samples collected from animal hosts. Several aspects of the molecular epidemiology and resistance development of *K. pneumoniae* in farm animal populations, wildlife, and foods need intensive research. Environmental health has to be integrated into national research plans, as a lack of data is apparent. Increasing awareness of the fact that non-human sources can act as a reservoir for this pathogen has to be raised.

## Introduction

Members of the genus *Klebsiella* (*K.*) belong to the family *Enterobacteriaceae*. They are Gram-negative, non-motile, usually capsulated, facultatively anaerobic bacteria. They are found in different environmental sources such as water and soil [1]. Up-to-date, the genus encompasses eight species: *K. pneumoniae*, which includes three subspecies (subspecies *pneumoniae*, *ozaenae,* and *rhinoscleromatis*); *K. oxytoca; K. planticola; K. ornithinolytica; K. granulomatis; K. mobilis, K. terrigena* and *K. variicola* [2]. *Klebsiella pneumoniae* is responsible for most human infections and one of the most critical multi-drug resistance (MDR) microorganisms worldwide [3]. The pathogen was found in the digestive, urinary, and respiratory tract of humans and can cause septic infection [4]. It is also found in a variety of environmental sources such as soil, water, and vegetation. It is often present in a wide range of domestic and wild mammals as well as in insects and has been also recovered from foods [5]. In animals, it is an essential cause of pneumonia, epidemic metritis, and cervicitis in mares and septicemia in foals [6]. It has been frequently associated with pneumonia and mastitis in bovines [7] leading to high losses in milk production, decreased milk quality, and even high mortalities among affected cows [8]. Consequently, infection can result in noticeable economic losses in the dairy industry, even in well-managed dairy farms [9]. However, its prevalence is increasing in dairy herds as well as in the food chain, research focusing on *K. pneumoniae* still very rare in veterinary medicine, and the risk of human infection after animal contact and food consumption is not well studied at all. Little information is available on the impact of *K. pneumoniae* infections on livestock welfare and productivity, epidemiology, resistance profiles, and resistance development in isolates of non-human sources.

Rapid dissemination and thus the rate of isolation of MDR *K. pneumoniae* strains are increasing in humans in Europe. In Germany, this trend is seen since the late twentieth century. Several strains with diverse antimicrobial resistance (AMR) gene patterns were isolated from several German states [10]. According to data from the Antibiotic Resistance Surveillance System (ARS), the European Antimicrobial Resistance Surveillance Network (EARS-Net) system and Surveillance of Antibiotic Use and Resistance in Intensive Care Units (SARI), the prevalence of MDR *K. pneumoniae* is dramatically increasing over the past few years in the human population [11]. Also the seriousness of the clinical outcomes is increasing over time [12].

## Background

### Factors relevant to the pathogenicity of *Klebsiella pneumoniae*

*Klebsiella pneumoniae* utilizes a variety of virulence factors. Although several genes involved in *K. pneumoniae* pathogenesis have been identified, their role in virulence [13] and resistance [14] is not entirely understood, as research only just begun. Four main components have long been associated with the pathogenesis of *K. pneumoniae*: K-capsular antigens, adherence factors, O-lipopolysaccharide (LPS), and siderophores [2]. Capsular Polysaccharide (K-antigen) was the first virulence factor described for *Klebsiella*. This antigen forms a thick hydrophilic capsule and is responsible for the glistening and mucoid appearance of *K. pneumoniae* colonies on agar plates. Up-to-date, at least 78 K-antigen serotypes have been characterized called K1, K2, etc. [15]. The K-antigens play a significant role in protection against opsonophagocytosis and against killing by serum [2]. Previous studies in a mouse model showed that K1 and K2 serotypes were more virulent than others. However, not all serotypes have been tested in animal models yet [16, 17]. Adhesions are often hemagglutinins located on fimbriae that protrude on the surface of the bacterial cells and are responsible for hemagglutination (HA). *Klebsiella pneumoniae* produces two types of fimbrial adhesions, type 1 and type 3 fimbriae. The thick-channeled (type-1) fimbriae, which are responsible for D-mannose-sensitive hemagglutination (MS-HA) are expressed by 80% of all *K. pneumoniae* strains. Clinical strains produce these fimbriae more often than in environmental isolates [18]. Thin, non-channeled (type-3) fimbriae, cause "mannose-resistant, *Klebsiella*-like hemagglutination” (MR/K-HA) and it expressed in more than 85% of *K. pneumoniae* strains and is encoded by the *mrk* gene cluster [18]. Additional adhesions were found to play a role in pathogenesis such as the non-fimbrial 29 kDa adhesin *“CF29K.”* [19], and the novel fimbrial adhesin “KPF-28” [20]. These adhesions are responsible for adherence of *K. pneumoniae* to intestinal cells and human carcinoma cells, respectively. Lipopolysaccharide (LPS) is composed of lipid A, a core polysaccharide, and a side chain called the “O-antigen”. Nine types of O-antigen are distinguished in *K. pneumoniae* and play a significant role in protection against complement-mediated killing [21, 22]. O1 is the most common antigen and is linked to extensive tissue necrosis. Siderophores or iron-scavenging systems are small, high-affinity iron-chelating compounds secreted by microorganisms and taken up again after they have “collected” Fe ions. *Klebsiella pneumoniae* is able to induce four to six iron-repressible outer-membrane proteins during infection. Enterochelin and aerobactin are secreted to solubilize and import the required iron ions during infection [23]. Recently, a third siderophore encoded by the *Yersinia* high-pathogenicity island called “yersiniabactin" was shown to be also present in *K. pneumoniae*, but its role in pathogenesis is still unknown [24]. The production of yersiniabactin was demonstrated in 17.7% of *K. pneumoniae* strains isolated from blood cultures and urine in hospitalized patients in Munich [25]. Additionally, there are other potential virulence factors like hemolysins produced in rabbit blood agar [26], heat-labile, and heat-stable enterotoxin [27], a protein-tyrosine kinase and a phosphotyrosine-protein phosphatase [28] which may be involved in the synthesis of capsular polysaccharide. Still, different aspects of *K. pneumoniae* pathogenicity e.g. infectious dose and incubation period are unknown.

### Resistance mechanisms in *Klebsiella pneumoniae*

*Klebsiella pneumoniae* poses a public health concern because it is one of the ‘ESKAPE’ pathogens, the most common MDR pathogens worldwide encompassing six bacterial pathogens (*Enterococcus faecium, Staphylococcus aureus, Klebsiella pneumoniae, Acinetobacter baumannii, Pseudomonas aeruginosa,* and *Enterobacter* species). It evades antimicrobial action with a variety of mechanisms including enzymatic degradation or inactivation of antimicrobial compounds, changing of membrane permeability, and modifying the target site of antimicrobial compounds by mutation of bacterial proteins. German *K. pneumoniae* strains have developed and acquired a massive variety of extended-spectrum β-lactamase enzymes (ESBL), which inhibit β-lactam antibiotics such as penicillins, cephalosporins, and carbapenems [29]. Genes responsible for enzyme inactivation are often located on mobile genetic elements (MGE) and provide a risk of transfer to other bacteria [30]. ESBL strains resistant to penicillins and cephalosporins were recovered from horses, dogs and cats admitted to veterinary clinics, and from European mouflons [31–33]. Changes in membrane permeability to antimicrobial compounds occur due to increased efflux or reduced influx of these compounds. MDR* K. pneumoniae* produce e.g. AmpC *β*-lactamase accompanied by loss of OmpK35 and OmpK36 proteins [34]. First reported German carbapenem-resistant *K. pneumoniae* strains had developed resistance through the loss of the porin channel protein OmpK36 and increased expression of a tripartite AcrAB-TolC efflux pump [35]. The occurrence of carbapenem-resistant *K. pneumoniae* is scarcely reported in animal hosts, probably because carbapenems use is forbidden in veterinary medicine. A carbapenemase-producing strain harboring *bla*OXA-48 was isolated from dogs in 2013 [36]. This genotype was reported again in studies with samples collected between 2012 and 2016 from dogs, cats, guinea pigs, rats, rabbits, and mice [37]. Mechanisms identified for the alteration of bacterial proteins to modify the target site of drugs were activation of ribosome-protective proteins, methylation of the ribosomal binding site, and amino acid exchanges in target genes due to mutations as in the case of resistance to fluoroquinolone. *Klebsiella pneumoniae* isolates resistant to fluoroquinolones were reported in Germany since 2003 [38]. In 2018, two plasmid-mediated quinolone-resistant (PMQR) strains that carried native *oqx*AB genes were reported from a dog. These strains were collected in 2014 during a study on the characterization of quinolone resistance mechanisms in *Enterobacteriaceae* from companion animals in Europe. They had mutations in the quinolone resistance-determining regions (QRDR) of the GyrA and ParC genes [39]. It can be supposed that German isolates of *K. pneumoniae* can develop all three types of resistance mechanisms.

## Spatio-temporal distribution of *K. pneumoniae* in non-human reservoirs in Germany

The number of reservoirs for resistant bacteria is increasing in hospitals, the community, and livestock as well as in the environment [40]. Therefore, transmission pathways between humans, animal hosts, and the environment are currently a subject of active discussion. Monitoring and understanding the current situation of resistance development and epidemiology of this pathogen in animals, foods, and the environment is necessary to combat this public health threat.

### Search strategy

#### Data acquisition and extraction

In the present work, articles focusing on *K. pneumoniae* in Germany until September 2020 obtained through searches in PubMed, Scopus, and Web of science. Following search terms were used: *Klebsiella pneumoniae* in Germany + antimicrobial-resistant + animals + food + environment. Articles discussing isolation and resistance profiles of *K. pneumoniae* are included. Studies dealing with human cases and other *Klebsiella* species were excluded. Title and abstract analysis of each publication and the full text of selected articles were analyzed.

#### Data setting and analysis

From 618 articles found, 125 were investigated. Ninety-nine articles are related to human studies (data not shown), and only 26 articles about prevalence and resistance development of *K. pneumoniae* in non-humans sources were identified [animal hosts (n = 13), foods (n = 3), and environmental sources (n = 10)]. The articles were published in a period from 1985 until September 2020. Seventeen studies were discussing the resistance patterns of strains, while the other nine articles were dealing only with isolation. The information was categorized chronically from the latest to the oldest article according to the year of publication based on host, source, and origin of isolates, the number of strains in each study, resistance profile and resistance genes present, location and time of sampling. The full details of the literature search are shown in Tables 1 and 2.

**Table 1:** *Klebsiella pneumoniae* isolates circulating in animals and foods in the last decades in Germany.

Source of isolates	No. of isolates	Resistance pattern	Genes detected	Locality of samples	Year of sampling	Authors and year of report	Refs.
*K. pneumonia* in Animals hosts (13 studies)							
Pet, zoo and falconry birds	86	Resistant to amoxicillin-clavulanic acid (10.2%), piperacillin-tazobactam (27.5%), enrofloxacin (11.6%), doxycycline (31.6%), sulfonamides (26.6%) and trimethoprim-sulfamethoxazole (9.1%)	ND	Southern Germany	2007–2016	Steger et al. 2020	[44]
Broiler chicken	7	ESBL recovered from a defeathering machine, scalding water, and skin	Strains harboring *bla*SHV-1, *bla*SHV-2, *bla*SHV-27 and TEM-1a	ND	2015–2016	von Tippelskirch et al. 2018	[43]
Dogs, cats, guinea pigs, rats, rabbits, mice	86	Resistant to imipenem, ampicillin, piperacillin, amoxicillin-clavulanate combination, fluoroquinolones, and different rates to aminoglycoside (amikacin, tobramycin, and gentamicin) were determined	Carbapenemase producer harboring *bla*OXA-48, 56 ESBL strains, carrying *bla*CTX-M-15, one with *bla*CTX-M-27, two with DHA-1 and CMY-2 genes	Giessen	2012–2016	Pulss et al. 2018	[37]
Dogs	2	(PMQR) Plasmid-Mediated Quinolone Resistance producers	Strains carried native *oqx*AB genes, with mutations in the quinolone resistance-determining regions (QRDR) of the GyrA and ParC genes	ND	2014	de Jong et al. 2018	[39]
European mouflons (*Ovis orientalis musimon)*	One	MDR, ESBL and AmpC phenotype, resistant to ampicillin, amoxicillin/ clavulanate, cefalotin, cefotaxime, cefoperazone, ciprofloxacin, enrofloxacin, trimethoprim-sulfamethoxazole, chloramphenicol, florfenicol, susceptible to imipenem, gentamicin, streptomycin, tetracyclines, and colistin	Strains carried the ß-lactamase genes (*bla*SHV-11, *bla*OXA-1, and *bla*DHA-1), plasmid-mediated quinolone resistance (PMQR) gene *qnr*B55. Mutations in the QRDR regions of the GyrA and ParC genes were not seen	ND	2012–2013	Loncaric et al. 2016	[33]
Horses, dogs, cats	34	MDR, ESBL producers, resistance to trimethoprim/sulfamethoxazole, ampicillin, cefotaxime, cefotaxime, cefepime	Strains harbored *bla*CTX-M-15, *bla*CTX-M-1, *bla*CTX-M-2 and *bla*OXA-48, *bla*TEM-1, *bla*SHV-1 and PMQR	Giessen, Hesse	2009–2011	Schmiedel et al. 2014	[32]
Horses, dogs, cats	72	ESBL producers, Resistance to fluoroquinolones, gentamicin, tetracycline trimethoprim/sulfamethoxazole, and tobramycin	Strains harbored *bla*CTX-M-15, *bla*CTX-M-1, *bla*CTX-M-3, *bla*CTX-M-9, *bla*SHV-2, *bla*SHV-12, *bla*SHV-28 and *bla*TEM-1	27 towns	2008–2010	Ewers et al. 2014	[31]
Common free-living reptiles	27	Slowworm (13), Grass snake (7), and European adders (7) Resistant patterns not determined	ND	Island Hidden- see	2012	Schmidt et al., 2014	[48]
Wildlife birds	ND	Common among water rails spotted crakes and barn swallows Resistant patterns not determined	ND	Saxony, Hamburg Brandenburg, Mecklenburg-Vorpommern	2008–2009	Stenkat et al. 2014	[47]
Dogs	109	MDR, resistance to gentamicin, amikacin, tobramycin, levofloxacin, ciprofloxacin, tetracycline, and trimethoprim/sulfamethoxazole	Five carbapenemase-producing strains harbored *bla*OXA-48 and ESBL harbored *bla*CTX-M-15. *bla*TEM-1, *bla*SHV-28, *bla*OXA-1, *bla*OXA-2 and (PMQR) plasmid-mediated quinolone resistance genes were expressed	Hessia	2012	Stolle et al. 2013	[36]
Burmese python	one	Resistant patterns not determined	ND	Leipzig	ND	Schroff et al. 2010	[46]
Domestic pigs	ND	Resistant patterns not determined	ND	ND	ND	Schierack et al. 2007	[41]
Pigeons	62	Recovered from liver, lung, heart, kidneys, small and large intestine Resistant patterns not determined	ND	Various parts of Germany	2003–2004	Raue et al. 2005	[45]
*K. pneumonia* in foods (3 studies)							
Black tiger shrimp	13	ESBL and/or AmpC producers,	Strains harbored *bla*SHV (-1a, -2a, -27, -32, -62, -186, -187), *bla*CTX-M-9, *bla*DHA-1, *bla*TEM-1	Berlin	2015–2016	Vu et al., 2018	[54]
Milk	one	WGS of novel temperate phage KPP5665-2 Resistant patterns not determined	ND	Stuttgart	2016	Carl et al. 2017	[53]
Uncooked vegetables	9	MDR, resistance to ampicillin, streptomycin, cotrimoxazole, tetracycline, sulfamethazine, chloramphenicol, and gentamicin	Strains harbored *aac*(3)-IIc gene	Wernigerode/Saxony-Anhalt	ND	Boehme et al. 2004	[51]

ND: not determined.

**Table 2:** Occurrence and summary of the resistance profile of *K. pneumoniae* in environmental sources in Germany.

Source	No. of strains	Resistance pattern	Genes detected	Locality of Sample	Year of sampling	Authors and year of report	Refs.
*K. pneumonia* in environmental sources (10 studies)							
Wastewater from poultry slaughterhouses	51	ESBL-producing *K. pneumoniae* and Six colistin resistance	Strains harbored *bla*SHV-1, 2, 25, 27, and 28, combinations SHV-2-TEM-1b and SHV-27-TEM-52b and CTX-M-1 were detected. Three strains with *mcr*-1 were isolated	ND	December 2016–September 2018	Savin et al. 2020	[66]
Water/sediment samples	9	MDR-ESBL producing strains	Strains harbored *bla*SHV–28, *bla*CTX–M–15, *tet*(D), *cat*B3, *aac*(3)-IId, *str*A, *str*B, *fos*A, *ere*(A), *sul*1, *sul*2, *oqx*A, *oqx*B, *aac*(6′)Ib-cr, *qnr*S1, *dfr*A5 and *dfr*A14	Northern Germany	2017	Falgenhauer et al. 2019	[65]
Raw sewage	one	ESBL producing strain 182 (WGS of virulent phage PMBT1). Resistant patterns not determined	ND	Kiel	ND	Koberg et al. 2017	[64]
Washing machines, dishwashers	ND		β-Lactamase genes AmpC- and OXA-type were detected	North-Rhine-Westphalia	ND	Rehberg et al. 2017	[63]
Wastewater treatment plants	ND	Colistin resistance, tetracycline resistance and erythromycin resistance	Strains harbored *mcr*-1, *tet*M, *erm*B gene, ß-lactam (*bla*CTX-M-32, *bla*TEM, *bla*CTX-M, and *bla*CMY-2)	ND	ND	Hembach et al. 2017	[61]
Dust from Pig farm	2	ESBL, resistant to ampicillin, ampicillin/sulbactam, cefuroxime, trimethoprim/sulfamethoxazole, and cefotaxime; and intermediate to piperacillin/tazobactam and ceftazidime and susceptible to carbapenems	Strain harbored *bla*CTX-M-1-like and *bla*SHVnon-ESBL genes	Lower Saxony, North-Rhine-Westphalia	2013	Garcia-Cobos et al. 2015	[60]
Moth fly *Clogmia albipunctata*	3	Resistance to ampicillin and piperacillin, sensitive to amoxicillin-clavulanate, gentamicin, cefuroxime, cefotaxime, amikacin, tobramycin, cotrimoxazole, aztreonam, levofloxacin, ciprofloxacin, ceftazidime, imipenem, meropenem, piperacillin, tazobactam	ND	ND	2011–2012	Faulde and Spiesberge, 2013	[59]
Natural surface waters	62	Resistant patterns not determined	ND	Schleswig–Holstein	1997–1998	Podschun et al. 2001	[58]
Water	ND	Resistant pattern not determined	ND	Rhine-Neckar region	1982–1983, 1986	Mersch-Sundermann and Wundt 1987	[57]
Raw wastewater	ND	92.8% were resist to gentamicin and trimethoprim	ND	ND	ND	Stelzer et al. 1985	[56]

ND: not determined.

### The situation in animal hosts

#### *Klebsiella pneumoniae* in farm and companion animals

*Klebsiella pneumoniae* recovered from clinical samples collected from companion and farm animals i.e. dogs, cats, horses, rabbits and rats, and chicken and pigs, respectively. Investigations on the composition of the intestinal *Enterobacteriaceae* populations of healthy pigs revealed that *K. pneumoniae* was detected in 84% of the subgroup of mucosa-associated bacteria, but the resistance pattern of these strains was not determined [41]. MDR carbapenemase and ESBL-producing strains were isolated from samples of soft tissues, urinary tract infections, the respiratory tract, the genital tract, wounds, and feces of dogs, cats, and horses [31, 32, 36]. A total of 109 K*. pneumoniae* strains was isolated from dogs between June and October 2012 in Hessia. Five isolates from the same veterinary clinic were carbapenem-resistant and harbored *bla*OXA-48 genes. All strains were clonally related, co-expressed ESBL of the *bla*CTX-M-15 type, and harbored plasmid-mediated quinolone resistance genes [36]. In between October 2008 to March 2010, 72 ESBL producing *K. pneumoniae* subsp. *pneumoniae* strains were recovered from companion animals including horses admitted to 30 veterinary clinics in 27 different towns in Germany [31]. The clonal group carrying *bla*CTX-M-15 was the most predominant type, while the *bla*CTX-M-1 group was less frequent. It is worth to mention that *bla*CTX-M-15 is the most prevalent ESBL in *K. pneumoniae* and has recently emerged in humans [42]. It is considered a zoonotic agent of high relevance to humans and animals [32, 42]. Between 2009 and 2011, 34 MDR-ESBL producing strains were recovered from dogs, cats, and horses admitted to veterinary clinics in Giessen [32]. Interestingly, isolates from companion animals, horses, and humans shared the same characteristics: presence of ESBL, carbapenemase OXA-48 and plasmid-encoded quinolone resistance (PMQR) genes. It is speculated that this coincidence of common features might prove the active transmission and dissemination of MDR genes between humans and animal populations in Germany [32]. However, this assumption needs further proof. Investigation of urine samples collected from dogs in 2014 revealed the presence of two PMQR producer strains, which carried native *oqx*AB genes [39]. Carbapenemase producer strains harboring *bla*OXA-48 and ESBL producers harboring *bla*CTX-M-15 and *bla*CTX-M-27 were detected in samples collected from dogs, cats, rabbits, guinea pigs, and mice between June 2012 and December 2016 [37]. The apparent relatedness of strains from different clinics investigated in that study points to the spread of this clone via animal carriers between various clinics, eventually contaminating the clinical environments [37]. Recently, ESBL strains harboring *bla*SHV-1, -2, -27, and TEM-1a were cultured from the skin of chicken broilers and a defeathering machine on the same farm [43]. Investigation of bacteria in samples collected between 2007 and 2016 from pet birds, zoo birds and falconry birds in Southern Germany revealed 86 K*. pneumoniae* isolates. The strains were resistance to doxycycline (31.6%), piperacillin-tazobactam (27.5%), sulfonamides (26.6%), enrofloxacin (11.6%), amoxicillin-clavulanic acid (10.2%) and trimethoprim-sulfamethoxazole (9.1%) [44]. The full details of our literature search are shown in Table 1.

#### *Klebsiella pneumoniae i*n wildlife

*Klebsiella pneumoniae* strains were recovered from internal organs (liver, kidney, heart, lung, small and large intestine) of pigeons captured in various parts of Germany between 2003 and 2004 [45], from snakes (*Burmese python*) suffering from pneumonia at Leipzig zoo [46]. Strains were also cultured from cloacal and pharyngeal swabs collected between 2008 and 2009 from free-living birds e.g. water rails (*Rallus aquaticus*), spotted crakes (*Porzana porzana*) and barn swallows (Hirundo *rustica*) in Saxony, Hamburg, Brandenburg, and Mecklenburg-Vorpommern [47]. In another study, 27 isolates were recovered from apparently healthy free-living reptiles on the island of Hiddensee in northeastern Germany. Seven strains were cultured from European adders (*Vipera berus*), seven isolates from grass snakes (*Natrix natrix*), and thirteen isolates from slow worms (*Anguis fragilis*) [48]. None of the previous studies aimed to characterize the resistance patterns for the strains or look for the existence of resistant genes. In contrast, the resistance profiles of MDR ST/11 strains isolated from nasal and perineal swabs of European mouflons (*Ovis orientalis musimon*) were investigated. The strains were collected between 2012 and 2013 and were ESBL and had an AmpC phenotype. They also carried several ß-lactamases and non-ß-lactamases plasmid-mediated quinolone resistance (PMQR) genes [33] (Table 1). *Klebsiella pneumoniae* was reported recently as one of the etiological bacterial agents causing death among the exotic captive amphibian pet and zoo animals in Germany [49].

### The situation in foods

Since manure is brought from animal farming to field and green land, resistant bacteria may spread to plants and the environment. Foodborne bacteria are extensively studied, but research on *K. pneumoniae* is scarce. *Klebsiella pneumoniae* has been recovered from various food samples, but especially from fresh raw chicken meat [5] and fresh vegetables worldwide [50]. Only three studies discussing the existence of *K. pneumoniae* in the food chains in Germany were found. Screening of twenty uncooked vegetables of different kinds i.e. tomatoes, salads, carrots, cauliflower, mushrooms, etc. as well as sprouts samples from Wernigerode/Saxony-Anhalt revealed the presence of nine MDR *K. pneumoniae* strains in rocket salad and mung bean sprouts [51]. The strains were resistant to ampicillin, streptomycin, tetracycline, chloramphenicol, sulfamethazine, cotrimoxazole, and kanamycin. Interestingly, one strain isolated from the pre-enrichment culture of sprouts was resistant to gentamicin and harbored the *aac* (3)-IIa gene [51]. Consumption of seed sprouts is a growing in market as an alternative product overall Europa. Outbreaks with pathogenic bacteria caused by contaminated sprouts demonstrated the risk of disseminate to humans via this route [52]. In 2016, a *K. pneumoniae* strain was isolated from a German mastitis milk sample by the CVUA-Stuttgart, Baden-Wuerttemberg [53]. The presence of resistant strains in milk is a threat to vulnerable animals and humans. Between December 2015 and August 2016, 160 retail raw seafood samples i.e. white leg shrimp, black tiger shrimp, blue mussels, venus clams, razor shells, and cockles were collected from a market in Berlin. Most of animals were originally harvested abroad. Thirteen ESBL and AmpC-producing *K. pneumoniae* were isolated from black tiger shrimps. Two samples from products were originally from Vietnam, and one from Bangladesh, while the rest could not be traced back to their country/ies of origin [54]. The strains were harboring *bla*SHV-1a (− 2a, − 27, − 32, 62, − 186, and − 187), *bla*CTX-M-9, *bla*DHA-1, and *bla*TEM-1 [54]. The samples used in that study were collected at retail markets. Therefore, the bacteria and AMR genes of those samples could have different origin, including wholesale handling, retail handling in supermarkets, the washing process, distribution, and seafood shops. That study highlights the potential hazards associated with seafood containing ESBL and AmpC producing *K. pneumoniae* in Germany. The presence of multi-resistance bacteria in foods is of concern to public health. Recently, evaluation of antibiotic resistance dissemination by wastewater treatment plant (WWTP) effluents with different catchment areas in Germany revealed that the daily discharge of *K. pneumoniae* in food-producing impacted WWTP effluents is higher compared to communal and hospital-impacted WWTP effluents [55].

### The situation in the environment

*Klebsiella pneumoniae* can be found in a variety of environments such as soil, water, and vegetation. In Germany*, K. pneumoniae* strains resistant to gentamicin and trimethoprim were isolated continuously from raw wastewater [56]. From May 1982 to January 1983 and March 1986 to May 1986, *K. pneumoniae* were collected from water specimens from the Rhine and its affluxes in the Rhine-Neckar-Region. At that time, the use of this water as unprocessed drinking water, for bathing, and for agriculture purposes was not acceptable [57]. From November 1997 to June 1998, 208 natural surface waters samples were collected from 196 different sampling sites at streams, lakes, and the Baltic Sea in Schleswig–Holstein. Among 123 *Klebsiella* strains isolated, *K. pneumoniae* was the most common species (n = 62) [58]. From June 2011 to May 2012, three strains resistant to ampicillin and piperacillin were recovered from adult moth flies (*Clogmia albipunctata*) captured around hospitals [59]. In 2013, two multi-resistant ESBL-producing strains were found in dust samples collected from pig farms in the federal states of Lower Saxony and North Rhine-Westphalia (NRW) [60]. Both strains were resistant to ampicillin, ampicillin/sulbactam, cefuroxime, cefotaxime, and trimethoprim/sulfamethoxazole and harboring the *bla*CTX-M-1-like and *bla*SHV non-ESBL genes. Colistin resistant strains carrying the *mcr-*1 gene were found in higher abundances in wastewater treatment plants even at effluent sampling sites [61]. In addition to *mcr-*1, the strains were harboring tetracycline *tet*M and erythromycin *erm*B resistance genes, and ß-lactamase (*bla*CTX-M-32, *bla*TEM, *bla*CTX-M, and CMY-2) genes. It is worth to mention that colistin-resistant strains carrying the *mcr-*1 gene were isolated in the same year from human samples collected from a leukemia patient in April 2015 at Frankfurt am Main [62]. Strains harboring β-lactamase AmpC- and OXA-type genes were detected in domestic washing machines and dishwashers in North Rhine-Westphalia [63]. MDR ESBL- *K. pneumoniae* strains were recovered from a sewage plant located in Kiel, northern Germany [64]. Hence, opportunistic pathogens and clinically relevant antibiotic resistance genes in wastewaters bear the risk of dissemination to the aquatic environment and from there to humans. Recently, hypervirulent *K. pneumoniae* isolates of four different sequence types (ST268, ST307, ST2155 and ST 3681) were isolated from water and sediment samples collected from water treatment plants in the neighborhood of a slaughterhouse in northern Germany. The strains were MDR and harbored *bla*SHV–28, *bla*CTX–M–15, *tet*(D), *cat*B3, *aac*(3)-IId, *str*A, *str*B, *fos*A, *ere*(A), *sul*1, *sul*2, *oqx*A, *oqx*B, *aac*(6′)Ib-cr, *qnr*S1, *dfr*A5 and *dfr*A14 [65]. These findings indicate the possible spread of MDR *K. pneumoniae* into the animal population. Fifty-one ESBL-producing *K. pneumoniae* strains were isolated from wastewater effluents of two poultry slaughterhouses between December 2016 and September 2018. The strains harbored the *bla*SHV-1, 2, 25, 27, and 28, a combinations of *bla*SHV-2-TEM-1b and *bla*SHV-27-TEM-52b, and CTX-M-1. Among them, six colistin resistance strains were identified, and *mcr*-1 was detected only in three [66]. The full details of our literature search are shown in Table 2.

## Discussion

Over the last decades, an alarming worldwide increase of MDR in *K. pneumoniae* strains isolated from humans has been noted, while the prevalence in wildlife species, farm and companion animals and the environment is not significantly investigated. The presence of MDR *K. pneumoniae* in animals and foods poses three significant problems. Firstly, the treatment of infection in animals will be challenging as the bacteria are resistant to various antibiotics approved for veterinary medicine. Secondly, the development of MDR strains in animal hosts and along the food chain may result in the development of reservoir with contamination or infection of humans finally. Thirdly, genes responsible for resistance are mainly plasmid-mediated and often located on mobile genetic elements (MGE) [30], which favors spread to obligate pathogens. Lateral gene transfer of a gene homologous of *ram*R of *Salmonella enterica* were identified in five isolates of *K. pneumoniae* resulting in reduced susceptibility to tigecycline [67]. *Klebsiella pneumoniae* is a common cause of bovine pneumonia, metritis, and mastitis. It can be assumed that milk can be easily contaminated during milking. The prevalence is increasing in dairy herds as well as in the food chain [68]. However, it is neglected in companion and other food-producing animals in Germany, and the risk of human infection concerning animal contact and food consumption is not well investigated. The present study highlights that MDR *K. pneumoniae* harboring several AMR genes are present in samples from animals, foods and the environment. For One-Health, these results are alarming and pin point the risk for the dissemination of resistance genes between animals, the environment, and healthcare professionals. New strategies are needed to control and prevent the evolution of MDR *K. pneumoniae* in veterinary hospitals i.e. reducing exposure of animals to antibiotics in the veterinary medicine. It is also crucial to screen and implement hygiene strategies to minimize spread. Collaboration between veterinary and public health professionals to combat antimicrobial resistance is supreme.

*Klebsiella pneumoniae* has been recovered from domestic animals, wildlife, the environment, and foods. Hence, only a few studies (n = 26) investigating *K. pneumoniae* in those non-human reservoirs were done from 1985 to September 2020. The *bla*CTX-M-15 and *bla*OXA-48 were the most predominant ESBL-genes in samples collected from companion animals and horses, and these are also the genes most often associated with antibiotic resistance in isolates of humans origin in Germany [32, 42]. The clonal group carrying *bla*CTX-M-15 can be considered a zoonotic agent of high relevance. Strains recovered from animal and human samples were found to share the presence of PMQR and OXA-48 genes, highlighting possible dissemination and transmission of the MDR genes between human and animal populations [32]. The hypervirulent *K. pneumoniae* ST268 was frequently associated with human disease and has been isolated from different surface waters in Northern Germany [65]. Colistin resistant strains carrying the *mcr*-1 gene was reported in 2015 in samples obtained from humans [62] and also detected at a higher rate in wastewater treatment plants [61]. The use of colistin in the treatment of MDR bacterial infection is continually increasing, resulting in emerging of colistin resistance *K. pneumoniae* in several countries worldwide and Europe [69].

As stated in the antibiotic resistance and consumption report (GERMAP 2015 report) which concern with the consumption of antimicrobials and the spread of antimicrobial resistance in human and veterinary medicine in Germany, *K. pneumoniae* is one of the main pathogens causing environmental mastitis in cattle. Since 2005/2006, the National Resistance Monitoring of Animal Pathogenic Bacteria (GERM-Vet) try to monitor *Klebsiella* spp. isolates from cows, and the 2013 study year included 39 strains. Comparing the results of the study years revealed that the resistance of *Klebsiella* were in an acceptable “not too high” range. However, the development of ESBLs requires special monitoring to be able to anticipate the trend in resistance development. The GERM-Vet report concluded that isolates from the "udder" compartment in dairy cattle showed significantly more favorable susceptibility levels than human strains in respect to antimicrobials used in veterinary medicine [70]. According to the antimicrobial surveillance report launched by the European Centre for Disease Prevention and Control (*ecdc*) in 2015, combined resistance to third-generation cephalosporins, fluoroquinolones, and aminoglycosides was prevalent in more than a third of the *K. pneumoniae* isolates reported to EARS-Net. However, this finding did not remain significant in Germany because only data from laboratories reporting consistently for 2012 to 2015 has been considered. The situation in neighboring EU countries is not much different. *Klebsiella pneumoniae* was extensively investigated in humans, but very few studies have been carried out on animals and foods. Between 2013 and 2014, PMQR-containing *K. pneumoniae* strains were isolated from dogs in Belgium, Spain, Poland, the Czech Republic [39], and *K. pneumoniae* was representing 2.3% of 7,806 bacterial isolates recovered from diseased equines in France between 2016 and 2019 [71]. Strains harbouring *bla*CTX-M-1, *bla*OXA-1, *qnr*B, *aac*(6), *aac*(6)-Ib, *cat*B3, *bla*SHV, *qrn*B, *qnr*S, *aph*A, *sul*1 and *dfr*A12 were isolated from dogs in Austria [72]. Comparative analysis of the epidemiology of *K. pneumoniae* in the EU countries and building up a network of researchers from these countries to investigate AMR in *K. pneumoniae* will greatly improve AMR surveillance.

## Conclusion

The current knowledge on the general distribution and antibiotic resistance in *K. pneumoniae* from domestic animals, wildlife, the environment, and foods in Germany is scarce. Food and environmental sources are playing a significant role in the transmission of antibiotic-resistant bacteria or their corresponding resistance determinants between animals and humans and from country to country. They are considered a concern for food and drinking water safety. The presence of MDR *K. pneumoniae* in foods, water and environment is alarming, and the potential health risks posed by such a way should not be underestimated. Increased awareness of public health and veterinary health is required. Further investigation of MDR pathogens in animals and the food chain is needed to clarify the transmission of AMR genes.

## Data Availability

All data are available in the tables.

## Abbreviations

AMR: antimicrobial resistance
ESBL: extended-spectrum β-lactamase
GERM-Vet: The National Resistance Monitoring of Animal Pathogenic Bacteria
HA: hemagglutination
*K*: *Klebsiella*
LPS: lipopolysaccharide
MDR: multi-drug resistant
MGE: mobile genetic elements:
MR/K-HA: "mannose-resistant, Klebsiella-like hemagglutination”
MS-HA: D-mannose-sensitive hemagglutination:
NRW: North Rhine-Westphalia
QRDR: quinolone resistance-determining regions
WWTP: Wastewater treatment plant
